# Supplementary figures and images for: Effect of virtual group counseling based on health literacy on the empowerment and self-care of pregnant women: A randomized controlled trial
Source: PLoS One. 2026 Feb 19;21(2):e0340706. doi: 10.1371/journal.pone.0340706 (PMC12919780; doi:10.1371/journal.pone.0340706)

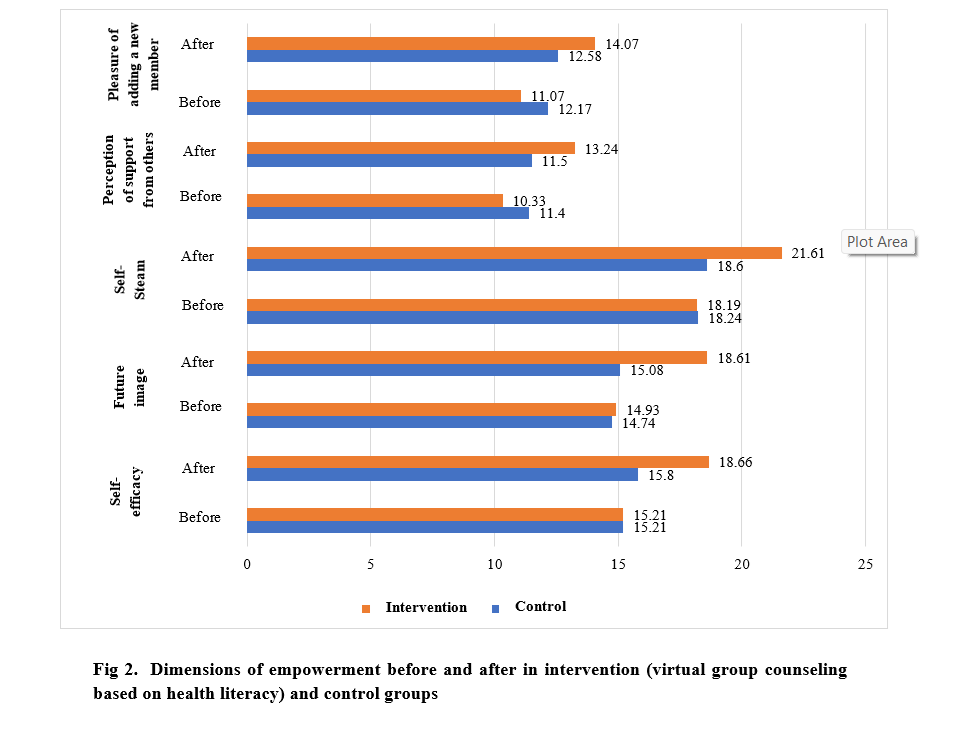

Supplement: S1 Fig — (TIF) [file pone.0340706.s001.tif]

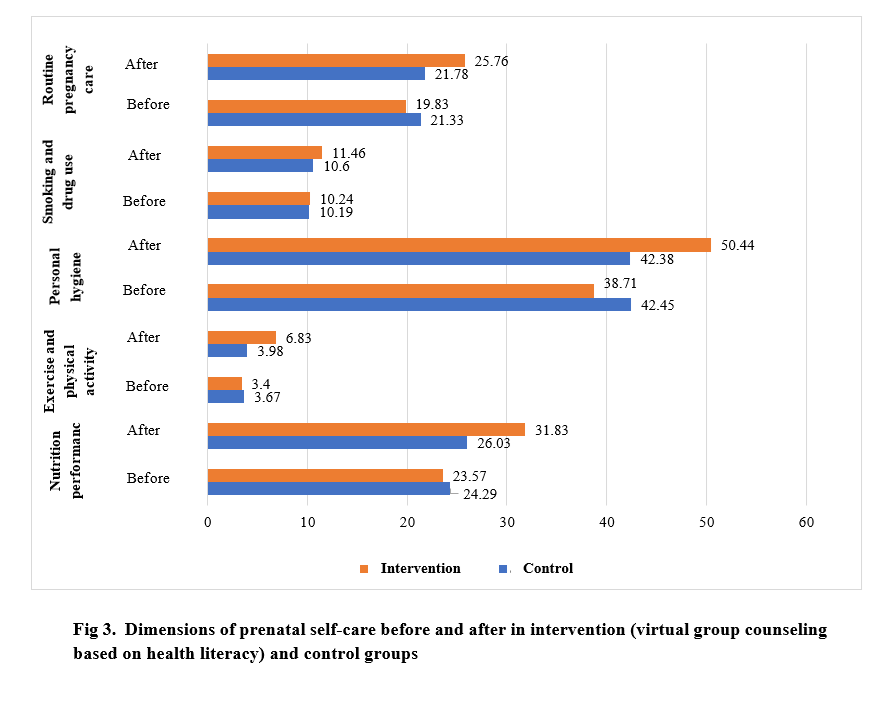

Supplement: S2 Fig — (TIF) [file pone.0340706.s002.tif]

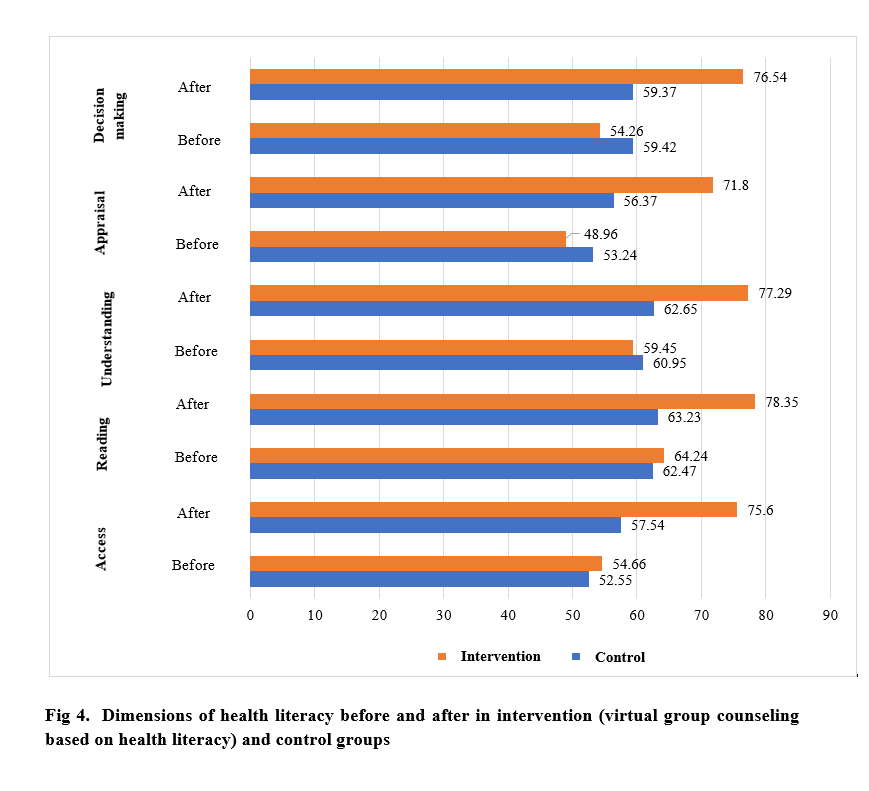

Supplement: S3 Fig — (TIF) [file pone.0340706.s003.tif]
