# Supplementary material for: Effect of virtual group counseling based on health literacy on the empowerment and self-care of pregnant women: A randomized controlled trial
Source: PLoS One. 2026 Feb 19;21(2):e0340706. doi: 10.1371/journal.pone.0340706 (PMC12919780; doi:10.1371/journal.pone.0340706)
Supplement: Attachment — Submitted filename: Response - 20251106.docx [file pone.0340706.s005.docx]

**Dear Editor**

I would like to thank the valuable comments of the respected reviewers; the following have been applied.

| **Comments** | **Response** |
| --- | --- |
| **Reviewer: 1** | |
| - Given that this research work has previously been published under a protocol in the journal BMJ Open with doi:10.1136/bmjopen-2024-097991, different parts of the manuscript should be reviewed and revised for any dissimilarity. | - Thank you for your valuable feedback. I have taken your suggestion into account and revised the manuscript to minimize dissimilarities with the previously published protocol (BMJ Open, doi:10.1136/bmjopen-2024-097991). Specifically, I reviewed and revised sections that could overlap, ensuring conceptual consistency while avoiding verbatim duplication, and I clearly delineated the methods and results to reflect the current study as an original full report. - I also removed Table 2. Outline of counseling intervention program which was in the protocol article. |
| - In Table 4, explain the details of the scores for the different dimensions of empowerment, self-care, and health literacy. | Table 4 has been changed to Table 3 in the new version. As per your valuable suggestion, the range of scores has been explained below the table. |
| Provide a brief explanation of the statistically significant relationship between the characteristics listed in Table 3 with Empowerment, Self-care, and Health literacy outcomes in the Methods part and a comparison with other studies in the Discussion part. | - It's done. |
| **Reviewer: 2** |  |
| - Introduction | - The entire text of the introduction was revised. |
| - Method Blinding Well-explained why full blinding is impossible. Could cite a methodological ref (e.g., Schulz & Grimes, Lancet, 2002) for handling blinding in behavioral RCTs. | - It's done. |
| - Discussion You mention “integration of health literacy principles” and “virtual mode increases access,” but mechanisms are not deeply discussed (e.g., cognitive engagement, social learning, self-efficacy theory). Needs theoretical depth. Implied in a few sentences (“integrating virtual counseling into routine care”), but not developed. Needs a closing paragraph connecting results to maternal health programs, virtual interventions, or health literacy policy. Only one brief mention (“further research needed to confirm long-term effects”). Should specify what kind of follow-up studies are recommended (e.g., longitudinal, multi-site, cost-effectiveness). | - It's done. |
