# Supplementary material for: Effect of virtual group counseling based on health literacy on the empowerment and self-care of pregnant women: A randomized controlled trial
Source: PLoS One. 2026 Feb 19;21(2):e0340706. doi: 10.1371/journal.pone.0340706 (PMC12919780; doi:10.1371/journal.pone.0340706)
Supplement: Attachment — Submitted filename: Response - 20251214.docx [file pone.0340706.s006.docx]

**Dear Editor**

I would like to thank the valuable comments of the respected reviewers; the following have been applied.

| **Comments** | **Response** |
| --- | --- |
| **Reviewer: 1** | |
| - Provide a brief explanation of the statistically significant relationship between the characteristics listed in Table 3 with Empowerment, Self-care, and Health literacy outcomes in the Methods part and a comparison with other studies in the Discussion part. It means analyze correlation between Characteristics listed in table (age, Level of education, job, family income…) with Empowerment, Self-care, and Health literacy scores in a table and Compare this correlation to other studies in the discussion part. | We thank the reviewer for this thoughtful comment. The primary objective of the present study was to evaluate the effect of a health literacy–based virtual group counseling intervention on empowerment, self-care, and health literacy outcomes within a randomized controlled trial framework. Accordingly, the sample size was calculated and powered exclusively to detect between-group differences in these outcomes, rather than to examine associations between baseline sociodemographic characteristics and outcome measures.  Moreover, as shown in Table 2 (changed number in new version), randomization resulted in well-balanced intervention and control groups, with no statistically significant differences in baseline sociodemographic or fertility characteristics. In randomized controlled trials, such baseline characteristics are not considered confounders, and conducting additional correlation or association analyses may be statistically underpowered and potentially misleading when the study is not designed for that purpose.  For these reasons, and in line with methodological recommendations for randomized trials, we did not perform additional correlation analyses between baseline characteristics and outcome measures. Instead, we focused on estimating the intervention effect using generalized estimating equations, which appropriately account for within-subject correlations over time and provide robust estimates of group-by-time interactions.  Importantly, the significant effects observed for empowerment, self-care, and health literacy can be attributed to the intervention itself rather than to baseline differences between participants. |
| - Explain the details of the scores for the different dimensions of empowerment, self-care, and health literacy: it means explain the details of the scores of empowerment domains such as self-efficacy, self-esteem, future image, and pleasure of adding a new member to the family, perception of support from others; and Self-care domains such as nutrition performance, personal hygiene, smoking and drug use, exercise and physical activity, and routine pregnancy care and also; five dimensions of Health literacy in the table. You haven't talked about the details of the domains' scores anywhere in the manuscript and have explained the scores in general. | It's done. Following this comment, figures 2, 3, and 4 were added to the findings and some points were also added to the discussion. |
